# Impact of PD-L1 Status on the Development of Cutaneous Immune-Related Adverse Events in Non-Small-Cell Lung Cancer Patients Receiving Immunotherapy

**DOI:** 10.3390/life16040636

**Published:** 2026-04-09

**Authors:** Alexander Yakobson, Fahed Abu Salamah, Ibrahim Alatawneh, Ahron Yehonatan Cohen, Yuliya Valdman-Grinshpoun, Yotam Malek, Itamar Gothelf, Ashraf Abu Jama, Nashat Abu Yasin, Mhammad Abu Juda, Arina Soklakova, Amichay Meirovitz, Walid Shalata

**Affiliations:** 1 The Legacy Heritage Center and Dr Larry Norton Institute, Soroka Medical Center, Beer Sheva 84105, Israel; 2 Faculty of Health Sciences, Ben Gurion University of the Negev, Beer Sheva 84105, Israel; 3 Department of Dermatology and Venereology, Soroka Medical Center, Beer Sheva 84105, Israel; 4 Goldman Medical School, Faculty of Health Sciences, Ben Gurion University of the Negev, Beer Sheva 84105, Israel

**Keywords:** non-small-cell lung cancer, immunotherapy, immune checkpoint inhibitors, overall survival, progression-free survival, real-world data, programmed cell death protein 1

## Abstract

**Background:** Cutaneous adverse events (cAEs) are among the most frequent immune-related toxicities associated with immune checkpoint inhibitors (ICIs). Previous clinical trials have reported higher rates of dermatologic toxicity with anti-CTLA-4 agents compared to Programmed Cell Death (PD-1)/Programmed Cell Death-1 Ligand (PD-L1) inhibitors. However, real-world data may differ due to evolving clinical experience and improved AE management strategies. **Methods:** We conducted a retrospective analysis of patients treated with ICIs to assess the incidence and severity of cAEs—specifically rash and pruritus—across different treatment regimens and PD-L1 expression subgroups, 285 patients treated with ICIs at Soroka Medical center during the years August 2018–November 2025. **Results:** Regarding dermatologic toxicity, 57 out of 285 patients (20%) experienced a rash. Among them, 46 patients (16% of total; 81% of those with rash) had grade 1, 7 patients (2% of total; 12% of those with rash) had grade 2, and 4 patients (1% of total; 7% of those with rash) had grade 3 reactions. No grade 4 or life-threatening cases were observed. Additionally, 47 patients (16.5%) developed pruritus, all grade 1–2. When stratified by treatment type, PD-L1 expression and the occurrence of immune-related adverse events, specifically rash and pruritus, were significantly associated with survival outcomes (*p* < 0.001). Patients with expressions of PD-L1 ≥ 1% had longer median overall survival (34.0 months) compared to those with expressions of PD-L1 < 1% (20.0 months), and longer progression-free survival (22.0 vs. 13.0 months). When considering rash, overall survival ranged from 19.0 months (PD-L1 < 1% with rash) to 36.0 months (PD-L1 ≥ 1% without rash), and progression-free survival ranged from 12.0 to 27.0 months. The presence of pruritus was associated with the most favorable outcomes, with median overall survival reaching 48.0 months and progression-free survival 31.0 months in patients with PD-L1 ≥ 1% and pruritus. All comparisons showed statistically significant differences (*p* < 0.001). **Conclusions:** These findings highlight that higher PD-L1 expression and the presence of immune-related adverse events—particularly pruritus—may serve as important prognostic indicators and could help inform personalized treatment strategies. The incidence and severity of cAEs in our study were consistent with prior clinical trials. The low frequency of grade ≥ 3 events may reflect increased familiarity with ICIs, leading to earlier recognition of adverse events, better patient education, and more effective management of skin toxicities.

## 1. Introduction

Lung cancer is a diverse disease that comprises many different variants. Lung cancer is primarily divided into two large groups: small-cell lung cancer (SCLC), which histologically is composed of neuroendocrine cells; and non-small-cell lung cancer (NSCLC), which is further sectioned based on its histology. NSCLC is mainly subdivided into squamous-cell carcinoma (SCC), adenocarcinoma, and large-cell carcinoma. An adenosquamous subtype has also been observed. NSCLC accounts for approximately 85% of all lung cancer cases, of which, the majority are classified as either squamous-cell carcinoma, or adenocarcinoma. Smoking is regarded as a major causative factor in the etiopathogenesis of all lung cancers. Nevertheless, adenocarcinoma is the most common type reported in non-smokers. Lung cancer is a leading cause of mortality worldwide, with late diagnosis contributing to its poor prognosis. Therefore, early diagnosis, with accurate tumor analysis, plays an integral role in establishing a proper treatment [1,2,3].

Across the different approaches of treating lung cancer, immunotherapy has become a promising modality. By integrating immune checkpoint inhibitors (ICIs), immunotherapy modulates and antagonizes the interaction between neoplastic and cytotoxic T-cells by utilizing targeted monoclonal antibodies. ICIs have emerged as a critical breakthrough in oncology, and are now broadly implemented in clinical practice [4,5].

ICI therapy may target the PD-1/PD-L1 pathway. PD-1 and PD-L1 are proteins which are expressed on tumor cells and cytotoxic T-lymphocytes, respectively. The interaction between PD-1 and PD-L1 enables tumor cells to bypass recognition by the immune system. Disrupting this interaction, is a fundamental aspect of ICIs therapeutic implementation [4,5,6].

The expression level of PD-L1, which is measured by immunohistochemistry, has been shown to be a predictive biomarker, with its expression level being an important factor. PD-L1 Tumor Proportion Score (TPS) may be reported as high expression (≥50%) or low expression (<50%), although other cutoffs can be made based on varying and supplementary thresholds (i.e., negative at <1%, intermediate at 1–49%). ICI therapy (e.g., pembrolizumab (Keytruda™) in patients with higher expression levels of PD-L1 has yielded positive results with better progression-free and overall survival in various types of malignancies [6,7,8,9,10].

Immunotherapy, despite its promise, has unfortunately been linked to immune-related adverse events (irAEs). Adverse effects of ICI treatment are notably common, and range from mild (grades 1–2) to severe (grade 3 and above). Virtually any organ system can be affected. Common ICI-related irAEs include, but are not limited to, cutaneous (rash, pruritus), gastrointestinal (diarrhea, colitis), endocrine (hypothyroidism, hyperthyroidism), and musculoskeletal (muscle and joint pain). Less frequently, neurological and cardiac toxicities have been observed. While most irAEs resolve, less common manifestations may be permanent or contribute to long-term sequelae [11,12,13,14].

Cutaneous irAEs are the most common adverse effects associated with ICI therapy. They are typically the first to manifest. Cutaneous irAEs vary widely, and may present as pruritus, xerosis, maculopapular or psoriasiform eruptions, depigmentation and alopecia. Although most irAEs are usually mild (low grade), more severe and rarer irAEs, such as Stevens–Johnson syndrome (SJS)/toxic epidermal necrolysis (TEN), drug reaction with eosinophilia and systemic symptoms (DRESS), and bullous diseases, can still occur. Therefore, recognizing cutaneous irAEs is essential for providing optimal care, which may involve discontinuation of ICI agents or the initiation of immunosuppressive therapy [14,15,16,17].

The mechanisms underlying cutaneous irAEs are complex and poorly understood, but some plausible explanations may include: reduced central tolerance of CD4+ and CD8+ T-cells targeting neoplastic and normal tissues, activation of autoreactive B-lymphocytes inducing cytotoxic effects, abnormal and dysregulated discharge of pro-inflammatory mediators, specific human leukocyte antigen (HLA) variations, and accelerated concurrent drug-induced eruptions. An increased incidence of cutaneous irAEs has been linked to improved prognosis and longer survival. This is thought to stem from an enhanced anti-tumor response. Thus, irAEs manifestations may serve as a vital indicator of therapy effectiveness and outcomes [16,17,18].

The objective of our study was to evaluate the impact of PD-L1 expression on the occurrence of cutaneous immune-related adverse events in patients with NSCLC undergoing immunotherapy, and to assess its influence on survival.

## 2. Materials and Methods

### 2.1. Study Population

This retrospective, single-center study examined a cohort of patients diagnosed with stage IV NSCLC. The treatment regimens under investigation included ICIs, specifically pembrolizumab or the combination of ipilimumab and nivolumab. These therapies were administered either as monotherapy or in combination with chemotherapy at the Soroka University Medical Center (SUMC) oncology department between 1 August 2018, and 1 November 2025. The non-squamous patients began treatment with a platinum-based chemotherapy agent—either cisplatin (75 mg/m^2^) or carboplatin (AUC 4–6)—in combination with pemetrexed (500 mg/m^2^). Based on the selected immunotherapy protocol, ICIs were added and continued as maintenance. Patients either received two cycles of chemotherapy with nivolumab (360 mg every three weeks) and ipilimumab (1 mg/kg every six weeks), followed by maintenance with pemetrexed, nivolumab, and ipilimumab; or they received pembrolizumab (200 mg every three weeks) alongside four cycles of chemotherapy, followed by maintenance with pemetrexed and pembrolizumab. Patients with squamous-cell carcinoma received intravenous cisplatin (75 mg/m^2^) or carboplatin (AUC 4–6), based on performance status, combined with paclitaxel (175 mg/m^2^). Immunotherapy included either nivolumab (360 mg every three weeks) with ipilimumab (1 mg/kg every six weeks) for two cycles, followed by maintenance with both agents, or pembrolizumab (200 mg every three weeks) administered for four cycles with chemotherapy, followed by pembrolizumab maintenance after discontinuation of the platinum and paclitaxel agents. All patients were treated according to the NCCN Clinical Practice Guidelines in Oncology [19].

### 2.2. Evaluation of Cutaneous Immune-Related Adverse Events (irAEs)

CirAEs were assessed using patient data from electronic health records, gathered either at the onset of ICI therapy or during the treatment course. These irAEs were graded according to the Common Terminology Criteria for Adverse Events (CTCAE) version 5, ranging from 1 to 5: Grade 1 (mild), Grade 2 (moderate), Grade 3 (severe), Grade 4 (life-threatening), and Grade 5 (fatal due to toxicity) [15,20]. The aim of our study was to investigate the association between PD-L1 expression and the development of cirAEs in patients with NSCLC receiving immunotherapy, while describing event grades and evaluating survival outcomes.

### 2.3. Statistical Analysis

Descriptive statistics were used to summarize the baseline demographic, clinical, and molecular characteristics of the patients. Continuous variables were assessed for normality using the Shapiro–Wilk test. Because these variables were not normally distributed, they were presented as median (range) and compared using the Mann–Whitney U test, while categorical variables were expressed as frequencies (percentages). To compare patient characteristics across different ICI groups, categorized according to the CTCAE scale, we employed either Fisher’s exact test or Pearson’s chi-square test, depending on the nature of the data. Kaplan–Meier survival analysis was conducted to evaluate overall survival (OS) and progression-free survival (PFS) across various Cutaneous irAEs categories. These analyses were further stratified by key clinical factors, including PD-L1 expression levels (PD-L1 was assessed by immunohistochemistry (IHC) using the 22C3 pharmDx assay (Agilent Technologies, Santa Clara, CA, USA) [21], chemotherapy use, type of ICI, gender, histological diagnosis, and age. Log-rank tests were performed to assess the statistical significance of differences in survival distributions. Multivariable analyses of PFS and overall survival OS were performed using Cox proportional hazard regression models to estimate hazard ratios (HRs) and their corresponding 95% confidence intervals (CIs). A two-sided significance level was set at *p* < 0.05 for all statistical tests. All analyses were conducted using SPSS software, version 29.0. In addition, the test that used for tables was “Pearson chi-square test”.

### 2.4. Study Design

The primary aim of this study was to evaluate the relationship between PD-L1 expression levels and the occurrence of cutaneous irAEs. Additionally, we sought to investigate whether PD-L1 expression and the occurrence of cutaneous irAEs were associated with treatment efficacy and survival outcomes in NSCLC patients receiving ICIs as first-line therapy. The primary outcome was the occurrence of cutaneous irAEs in relation to PD-L1 levels, while secondary outcomes included OS and PFS. OS was defined as the time from the initiation of ICI therapy to death from any cause, with patients alive at the last follow-up being censored. PFS was defined as the period from the first dose of ICI until the earliest of disease progression (based on radiologic confirmation) or death. Disease progression was assessed using the Response Evaluation Criteria in Solid Tumors (RECIST) version 1.1. [22] To provide a comprehensive understanding of the cohort, we first described the patients’ characteristics across different groups. Univariate analyses were conducted to explore associations between the type of immunotherapy and the outcomes. Multivariable analysis was performed, adjusting for potential confounders such as histologic subtype (adenocarcinoma and SCC), gender (female vs. male), age (<70 vs. ≥70 years), smoking status (never, current, or past smoker), Eastern Cooperative Oncology Group Performance Status (ECOG-PS; 0–1 vs. ≥2), treatment modality (immunotherapy alone versus combination chemo-immunotherapy), and, among patients receiving immunotherapy, the specific regimen (ipilimumab plus nivolumab versus pembrolizumab), and PD-L1 expression levels (<1%, 1–49%, and >50%).

## 3. Results

We identified 285 patients who were treated at our centers, all of whom had complete follow-up data, including documented adverse events. The results showing that the median age was 66.8 years, ranging from 41 to 86 years. Of the participants, 33.3% (*n* = 95) were female and 66.6% (*n* = 190) were male. Histologically, 66.7% (*n* = 190) of patients were diagnosed with adenocarcinoma, while 33.3% (*n* = 95) had squamous-cell carcinoma. Regarding smoking status, 13.3% (*n* = 38) of the patients were never smokers, 49.1% (*n* = 140) were current smokers, and 37.7% (*n* = 107) were past smokers. Performance status based on ECOG scores revealed that 20.7% (*n* = 59) had a score of 0, 59.6% (*n* = 170) had a score of 1, and 19.7% (*n* = 56) had a score of 2 or higher. In terms of treatment, 79% (*n* = 225) received chemo-immunotherapy, while 21% (*n* = 60) were treated with only immunotherapy. Among those receiving immunotherapy, 71.2% (*n* = 203) were administered pembrolizumab, and 28.8% (*n* = 82) received the combination of ipilimumab plus nivolumab. PD-L1 expression levels were available for all patients: 35.8% (*n* = 102) had PD-L1 expression < 1%, and 64.2% (*n* = 183) had expression > 1%. Within the group with PD-L1 > 1%, 21.7% (*n* = 62) had expression between 1–49%, and 42.5% (*n* = 121) had PD-L1 levels greater than 50%, (Table 1).

Regarding dermatological toxicity, we observed that 57 patients (20%) overall experienced rash. Among the 57 patients who experienced rash, 46 patients had grade 1, 7 patients had grade 2, and 4 patients had grade 3 reactions. No life-threatening cases (grade 4) were reported. In addition, 47 patients (16.5%) had pruritus all grade 1–2. When comparing these outcomes by PD-L1 status, we found that among patients with PD-L1 < 1%, 20 patients (19.6%) had a rash, while 37 patients (20.2%) with PD-L1 > 1% experienced rash. The difference between these groups was not statistically significant (*p* = 0.91) (Table 2). For the PD-L1 >1% subgroup, we found no statistically significant difference between the PD-L1 with staining of 1–49% and PD-L1 > 50% groups (*p* = 0.083). Specifically, 13 patients (7.1%) with PD-L1 staining 1–49% had a rash, compared to 24 patients (13.1%) with PD-L1 staining > 50%, (Table 2).

For pruritus, 9 patients (8.8%) with PD-L1 < 1% had this adverse effect, while 38 patients (20.2%) with PD-L1 > 1% experienced it, showing a significant statistical difference (*p* = 0.0013). However, when comparing the subgroups within PD-L1 > 1%, there was no significant difference between the PD-L1 staining 1–49% group (14 patients, 7.1%) and the PD-L1 staining ≥ 50% group (24 patients, 13.1%), (*p* = 0.206), (Table 2 used Pearson chi-square test).

When comparing dermatological adverse events between patients who received immunotherapy alone and those who received a combination of treatment (chemo-immunotherapy), there was no significant difference observed in the incidence of rash. Specifically, 11 patients (18.3%) out of 60 who received only immunotherapy developed a rash, compared to 46 patients (20.4%) out of 225 who received chemo-immunotherapy (*p* = 0.856), (Table 3). Similarly, for pruritus, no significant difference was found between the two treatment groups. Pruritus occurred in 11 patients (18.3%) in the immunotherapy-only group and in 35 patients (15.6%) in the chemo-immunotherapy group (*p* = 0.747), (Table 3, used Pearson chi-square test). Notably, all grade 2 and 3 cases occurred in patients receiving combination chemo-immunotherapy.

Comparison between the two immunotherapy regimens—pembrolizumab and ipilimumab plus nivolumab—showed that rash occurred in 13.4% (11 patients) of those receiving ipilimumab plus nivolumab and in 21.1% (43 patients) of those receiving pembrolizumab. This difference was not statistically significant (*p* = 0.181). Similarly, pruritus was reported in 9.8% (8 patients) of the ipilimumab plus nivolumab group compared to 18.7% (38 patients) in the pembrolizumab group, which also did not reach statistical significance (*p* = 0.075), (Table 4, used Pearson chi-square test).

When comparing rash incidence by gender, 22.1% of females (21 patients) experienced rash compared to 19% of males (36 patients), with no statistically significant difference (*p* = 0.535). Similarly, pruritus was reported in 22% of males (19 patients) and 14.2% of females (27 patients), which also did not show a statistically significant difference (*p* = 0.118), (Table 5, used Pearson chi-square test).

In terms of age at the onset of symptoms, patients who developed a rash had a median age of 68.6 years, while those without a rash had almost the same ages with median age of 66.4 years; this difference was statistically significant (*p* = 0.033). In contrast, the median age of patients with pruritus was 67.7 years compared to 66.7 years in those without, which was not statistically significant (*p* = 0.51).

**Figure 1:**
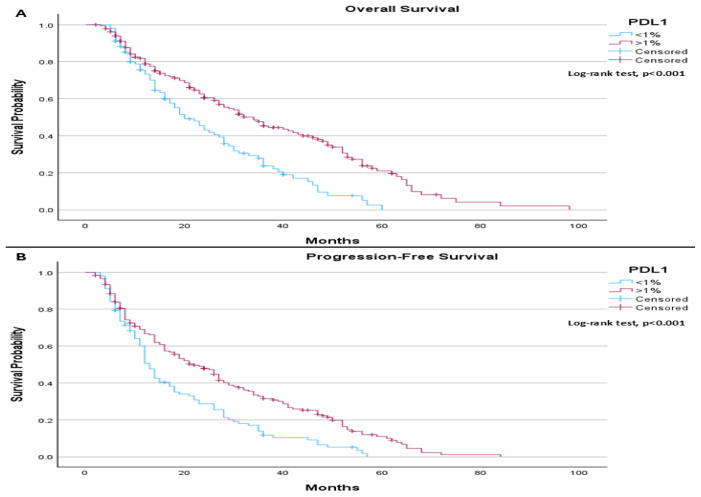
(**A**) Overall Survival: The median overall survival was 20.0 months (95% CI: 14.88 to 25.12) for patients with PD-L1 expression < 1%, and 34.0 months (95% CI: 27.40 to 40.60) for those with PD-L1 expression ≥ 1%. The log-rank test showed a statistically significant difference in overall survival between the groups (*p* < 0.001). (**B**) Progression-Free Survival: The median progression-free survival was 13.0 months (95% CI: 11.32 to 14.68) for patients with PD-L1 < 1%, and 22.0 months (95% CI: 16.55 to 27.45) for those with PD-L1 ≥ 1%. The log-rank test demonstrated a statistically significant difference in progression-free survival between the groups (*p* < 0.001).

Median follow-up was 26.4 months, when comparing median overall survival based on PD-L1 status, a statistically significant difference was observed between the groups (*p* < 0.001). Patients with PD-L1 expression < 1% (102 patients (35.8%)) had a median overall survival of 20.0 months, whereas those with PD-L1 expression ≥ 1% had a median overall survival of 34.0 months (Figure 1A). A similar statistically significant difference was found in progression-free survival (*p* < 0.001), with median values of 13.0 months for patients with PD-L1 < 1% and 22.0 months for those with PD-L1 ≥ 1% (183 patients (64.2%)) (Figure 1B).

When evaluating median overall survival in relation to both PD-L1 status and the occurrence of rash as an adverse event, a statistically significant difference was found among the groups (*p* < 0.001). Patients with PD-L1 < 1% with rash OS is better than for without >1% (22 months vs. 19 months). In contrast, those with PD-L1 expression ≥ 1% had a median overall survival of 36.0 months without rash and 28.0 months with rash. A similar statistically significant difference was observed in progression-free survival (*p* < 0.001). For patients with PD-L1 < 1%, the median progression-free survival was 12.0 months without rash and 18.0 months with rash. Among those with PD-L1 ≥ 1%, progression-free survival was 20.0 months without rash and 27.0 months with rash.

When assessing median overall survival based on PD-L1 status and the presence of pruritus as an adverse event, a statistically significant difference was observed among the groups (*p* < 0.001). Patients with PD-L1 expression < 1% had a median overall survival of 19.0 months without pruritus and 23.0 months with pruritus. In comparison, those with PD-L1 expression ≥ 1% had a median overall survival of 32.0 months without pruritus and 48.0 months with pruritus. Similar statistically significant differences were noted in progression-free survival (*p* < 0.001). Median progression-free survival was 12.0 months for patients with PD-L1 < 1% without pruritus, 23.0 months for those with PD-L1 < 1% with pruritus, 19.0 months for PD-L1 ≥ 1% without pruritus, and 31.0 months for PD-L1 ≥ 1% with pruritus.

**Figure 2:**
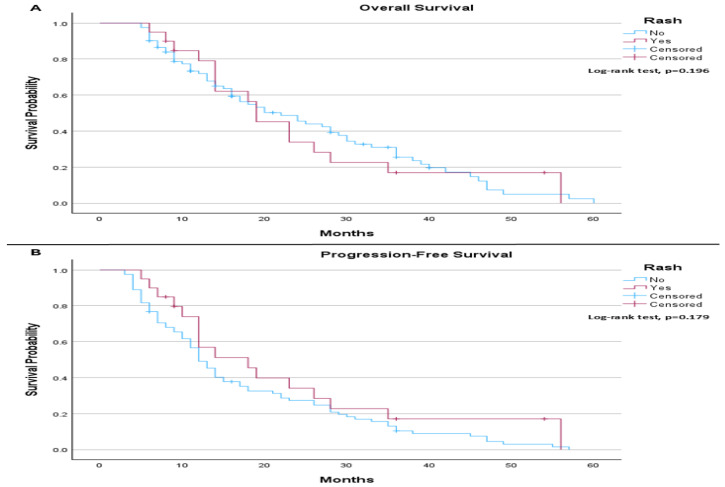
(**A**) Overall Survival (PD-L1 ≤ 1% patients only): The median overall survival was 22.0 months (95% CI: 14.50 to 29.50) for patients without rash and 19.0 months (95% CI: 13.91 to 24.10) for those with rash. The log-rank test did not indicate a statistically significant difference in overall survival between the groups (*p* = 0.196). (**B**) Progression-Free Survival (PD-L1 ≤ 1% patients only): The median progression-free survival was 12.0 months (95% CI: 9.995 to 14.005) for patients without rash and 18.0 months (95% CI: 8.561 to 27.439) for those with rash. The log-rank test did not demonstrate a statistically significant difference in progression-free survival between the groups (*p* = 0.179).

When comparing the survival outcomes according to rash and PD-L1 expression, the results show that, among patients with PD-L1 ≤ 1%, the median OS was 22.0 months in patients without rash compared to 19.0 months in those who developed rash. There was no statistically significant difference in OS between the groups (log-rank *p* = 0.196; Figure 2A). The median PFS was 12.0 months in patients without rash and 18.0 months in those with rash, without a statistically significant difference (log-rank *p* = 0.179; Figure 2B).

**Figure 3:**
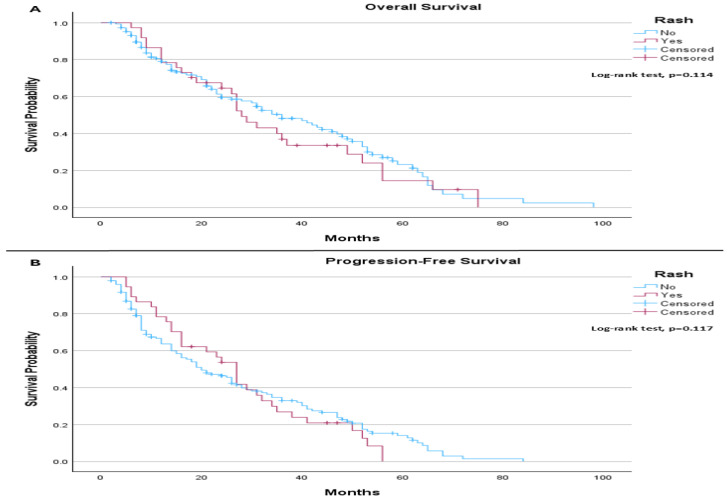
(**A**) Overall Survival (PD-L1 > 1% patients only): The median overall survival was 36.0 months (95% CI: 26.77 to 45.23) for patients without rash and 28.0 months (95% CI: 23.48 to 32.52) for those with rash. The log-rank test did not indicate a statistically significant difference in overall survival between the groups (*p* = 0.114). (**B**) Progression-Free Survival (PD-L1 > 1% patients only): The median progression-free survival was 20.0 months (95% CI: 14.24 to 25.76) for patients without rash and 27.0 months (95% CI: 22.59 to 31.41) for those with rash. The log-rank test did not demonstrate a statistically significant difference in progression-free survival between the groups (*p* = 0.117).

In the PD-L1 > 1% subgroup, the median OS was 36.0 months for patients without rash and 28.0 months for those with rash. The difference did not reach statistical significance (log-rank *p* = 0.114; Figure 3A). Median PFS was 20.0 months in patients without rash and 27.0 months in those with rash, with no statistically significant difference observed (log-rank *p* = 0.117; Figure 3B).

**Figure 4:**
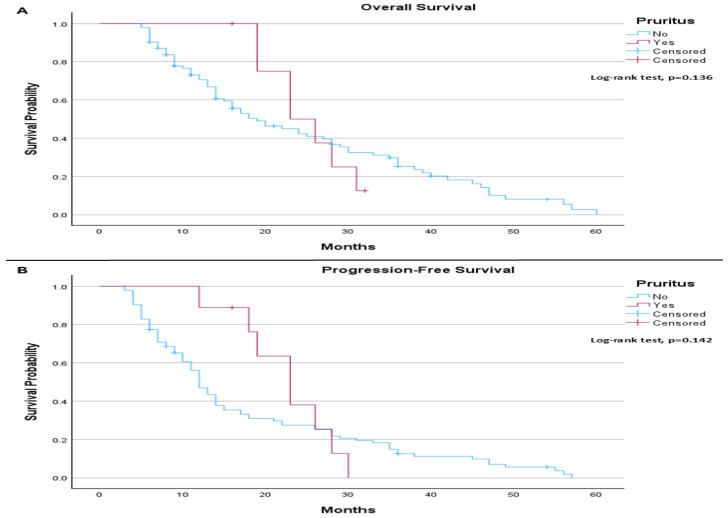
(**A**) Overall Survival (PD-L1 ≤ 1% patients only): The median overall survival was 19.0 months (95% CI: 12.88 to 25.12) for patients without pruritus and 23.0 months (95% CI: 16.53 to 29.47) for those with pruritus. The log-rank test did not indicate a statistically significant difference in overall survival between the groups (*p* = 0.136). (**B**) Progression-Free Survival (PD-L1 ≤ 1% patients only): The median progression-free survival was 12.0 months (95% CI: 10.36 to 13.65) for patients without pruritus and 23.0 months (95% CI: 17.68 to 28.32) for those with pruritus. The log-rank test did not demonstrate a statistically significant difference in progression-free survival between the groups (*p* = 0.142).

When comparing the survival outcomes according to pruritus and PD-L1 expression, we notice that, among patients with PD-L1 ≤ 1%, the median OS was 19.0 months in patients without pruritus and 23.0 months in those with pruritus. This difference was not statistically significant (log-rank *p* = 0.136; Figure 4A). Median PFS was 12.0 months in patients without pruritus and 23.0 months in those with pruritus, also without statistical significance (log-rank *p* = 0.142; Figure 4B).

**Figure 5:**
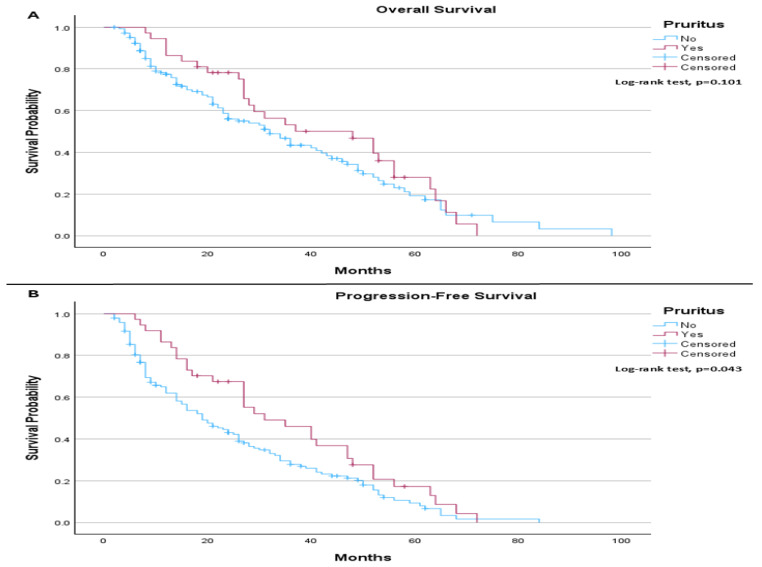
(**A**) Overall Survival (PD-L1 > 1% patients only): The median overall survival was 32.0 months (95% CI: 24.17 to 39.83) for patients without pruritus and 48.0 months (95% CI: 26.62 to 69.38) for those with pruritus. The log-rank test did not indicate a statistically significant difference in overall survival between the groups (*p* = 0.101). (**B**) Progression-Free Survival (PD-L1 ≥ 1% patients only): The median progression-free survival was 19.0 months (95% CI: 13.45 to 24.55) for patients without pruritus and 31.0 months (95% CI: 16.84 to 45.16) for those with pruritus. The log-rank test indicated a statistically significant difference in progression-free survival between the groups (*p* = 0.043).

In the PD-L1 > 1% subgroup, the median OS was 32.0 months in patients without pruritus compared to 48.0 months in those with pruritus, without reaching statistical significance (log-rank *p* = 0.101; Figure 5A). However, median PFS was 19.0 months in patients without pruritus and 31.0 months in those with pruritus, demonstrating a statistically significant improvement in PFS among patients who developed pruritus (log-rank *p* = 0.043; Figure 5B).

In the time-dependent Cox regression analysis for OS, neither rash nor pruritus demonstrated a significant association with OS. Rash was not associated with improved or worsened survival (HR 1.12, 95% CI 0.82–1.54, *p* = 0.47), and similarly, pruritus showed no statistically significant effect (HR 0.81, 95% CI 0.58–1.14, *p* = 0.23). In contrast, PD-L1 expression ≥ 1% was significantly associated with improved OS (HR 0.62, 95% CI 0.48–0.80, *p* < 0.001). Increasing age was associated with a modest but statistically significant increase in mortality risk (HR 1.02 per year, 95% CI 1.01–1.04, *p* = 0.021). Poor performance status (ECOG ≥ 2) was also strongly associated with worse OS (HR 1.78, 95% CI 1.29–2.45, *p* < 0.001), as was current smoking status (HR 1.34, 95% CI 1.01–1.78, *p* = 0.043). Male sex, squamous histology, and receipt of chemo-immunotherapy were not significantly associated with OS, (Table 6).

For PFS, rash was not significantly associated with outcomes (HR 0.97, 95% CI 0.74–1.28, *p* = 0.84). However, pruritus was significantly associated with improved PFS (HR 0.68, 95% CI 0.50–0.92, *p* = 0.014). PD-L1 expression ≥ 1% was again associated with improved PFS (HR 0.59, 95% CI 0.46–0.75, *p* < 0.001). ECOG performance status ≥ 2 remained a strong predictor of worse PFS (HR 1.65, 95% CI 1.23–2.20, *p* = 0.001). Age showed a non-significant trend toward worse PFS (HR 1.01 per year, 95% CI 1.00–1.03, *p* = 0.08), while current smoking also demonstrated a borderline association with poorer PFS (HR 1.28, 95% CI 0.98–1.66, *p* = 0.07). No significant associations were observed for sex, histology, or chemo-immunotherapy in relation to PFS, (Table 7).

## 4. Discussion

Although immunotherapy has revolutionized the management of lung cancer—particularly NSCLC—a considerable proportion of patients still fail to achieve meaningful clinical benefit. Evaluating true clinical response to ICIs remains challenging for clinicians, and irAEs often necessitate treatment discontinuation, especially with combination regimens. The broad spectrum of irAEs, affecting multiple organ systems, further complicates the recognition of therapy-related toxicities. These challenges may be amplified in settings with limited access to specialized oncology care, potentially delaying diagnosis and intervention.

Cutaneous toxicities are among the most frequently reported irAEs, accounting for over 50% of cases, and are generally mild, rarely necessitating treatment discontinuation. Nonspecific maculopapular rashes, often accompanied by pruritus, typically appear within the first six weeks of therapy, usually involving less than 30% of the body surface area, with grade ≥ 3 events occurring in fewer than 5% of patients. Lichenoid eruptions are more common under PD-1 inhibitors, appearing in up to 30% of patients, whereas CTLA-4 inhibitors are less frequently associated with these reactions. The pathophysiology of cutaneous irAEs remains incompletely understood, but several immune-mediated mechanisms have been proposed. One leading hypothesis is cross-reactivity, in which checkpoint inhibition enhances T-cell responses against antigens shared by tumor cells and normal skin. In NSCLC, antigen-specific T-cell clones have been identified in both tumor tissue and skin lesions, supporting this mechanism. In addition, PD-1 and CTLA-4 blockade may promote Th1/Th17-driven inflammation and increased cytokine production, contributing to manifestations such as maculopapular, lichenoid, and psoriasiform eruptions, as well as pruritus. These mechanisms may help explain why cutaneous irAEs are among the most common and earliest toxicities seen during ICI therapy [16].

In our cohort of 285 patients, dermatologic toxicities—specifically rash and pruritus—were observed in 20% and 16.14% of patients, respectively. Stratification by PD-L1 expression revealed statistically significant associations between PD-L1 status and the occurrence of these cutaneous events. Patients with PD-L1 ≥ 1% experienced longer median OS (34.0 months vs. 20.0 months) and PFS (22.0 vs. 13.0 months) compared to those with PD-L1 < 1%. When considering rash, OS ranged from 19.0 months (PD-L1 < 1% with rash) to 36.0 months (PD-L1 ≥ 1% without rash), while PFS varied from 12.0 to 27.0 months. Although rash was associated with a trend toward longer PFS, this did not translate into improved OS, suggesting that any association between rash and outcome should be interpreted cautiously given the small subgroup size and potential confounding factors.

Pruritus demonstrated an even stronger association with outcomes: patients with PD-L1 ≥ 1% who developed pruritus had the longest median OS (48.0 months) and PFS (31.0 months), compared to 19.0 and 12.0 months, respectively, for PD-L1 < 1% patients without pruritus.

Although the incidence of rash was only slightly higher in PD-L1 ≥ 1% patients (20.2%) compared to PD-L1 < 1% (19.6%; *p* = 0.91), pruritus occurred significantly more frequently in the PD-L1 ≥ 1% group (20.2% vs. 8.8%; *p* = 0.0013), supporting the hypothesis that higher PD-L1 expression may be associated with increased immune activation and skin toxicity. Subgroup analysis within PD-L1-positive patients (1–49% vs. >50%) did not reveal significant differences in rash (*p* = 0.083) or pruritus (*p* = 0.206), suggesting that PD-L1 positivity per se may influence dermatologic toxicity risk, but finer stratification does not substantially alter incidence.

No significant differences in cutaneous toxicities were observed between combination therapy (ipilimumab plus nivolumab) and monotherapy (pembrolizumab), despite prior reports suggesting higher rates with anti-CTLA-4 agents. This may reflect the predominance of monotherapy in our cohort, limiting statistical power, or differences in patient characteristics, treatment duration, and early toxicity management strategies [23,24]. Similarly, rates of rash and pruritus were comparable between patients receiving immunotherapy alone and those on chemo-immunotherapy, acknowledging that chemotherapy-related skin reactions could be misclassified as irAEs.

Our observed incidence of rash (20%) and pruritus (16.5%) aligns with major clinical trials, including KEYNOTE-189 [23], KEYNOTE-407 [24], and CheckMate 9LA [25]. The low frequency of severe (grade ≥ 3) cutaneous events may be due to increased clinician experience with ICIs, proactive monitoring, early intervention, and patient education, which facilitate timely management and prevent progression to higher-grade toxicity [26,27,28,29].

For managing ICI-related cutaneous adverse events, treatment depends on the severity of the rash. In cases of grade 1 maculopapular rash, ICI therapy can generally be continued, with management focused on topical corticosteroids, oral antihistamines, emollients, and appropriate investigations to exclude other causes. For grade 2 toxicity, temporary interruption of ICI therapy should be considered, alongside the use of higher-potency topical steroids and supportive medications; systemic corticosteroids may be introduced if symptoms are refractory. Grade 3 rashes—characterized by involvement of more than 30% of the body surface area with moderate symptoms—require oral prednisone at 0.5–1 mg/kg in addition to topical and supportive treatments. Grade 4 toxicities are severe and potentially life-threatening, necessitating hospital admission and initiation of intravenous methylprednisolone at 1–2 mg/kg. When systemic steroids are used, they should be tapered gradually over at least four weeks once improvement is observed. For patients with grade 3 or higher toxicity, or those hospitalized, early dermatology consultation is essential to guide management and consider second-line therapies in cases of steroid-refractory toxicity [27,28,29].

This study has several limitations. First, its retrospective design introduces the potential for selection bias and limits the ability to establish causal relationships. Furthermore, retrospective analyses are also prone to confounding, particularly time-on-treatment and immortal time bias, since patients with longer survival inherently have a greater opportunity to develop and report adverse events.

Second, the sample size—particularly within specific subgroups such as patients receiving combination chemo-immunotherapy—may have been insufficient to detect small but clinically meaningful differences in the incidence of cutaneous adverse events. Moreover, because rash and pruritus were analyzed separately, potential overlap between these cutaneous events could not be evaluated. In addition, attribution of cutaneous toxicities in patients receiving chemo-immunotherapy is inherently challenging in real-world retrospective data. Although patients were analyzed according to treatment modality (immunotherapy alone vs. chemo-immunotherapy), chemotherapy-related skin reactions cannot be fully excluded and may have been misclassified as immune-related events in some cases. Third, the grading and reporting of dermatologic toxicities relied on routine clinical documentation using CTCAE criteria, which may be subject to underreporting, incomplete capture of low-grade events, and inter-observer variability among treating clinicians.

Finally, the study population was derived from a single center, which may limit the generalizability of the findings to broader and more diverse patient populations.

## 5. Conclusions

Our study demonstrates that cutaneous adverse events, primarily rash and pruritus, remain common but manageable toxicities among patients receiving immune checkpoint inhibitors. The incidence and severity of these dermatologic AEs in our cohort align closely with those reported in pivotal clinical trials. Importantly, the low occurrence of severe (grade ≥ 3) skin toxicities likely reflects increased clinical experience, improved early detection, and proactive management strategies developed with the expanded use of immunotherapy. Additionally, the higher frequency of rash and pruritus observed in patients with elevated PD-L1 expression highlights the need for careful monitoring in this subgroup. Overall, these findings emphasize the importance of vigilant assessment and timely intervention to minimize the impact of cutaneous toxicities and optimize patient outcomes during immunotherapy.

## Figures and Tables

**Table 1:** Demographics of patients participants in our cohort, *n* = 285.

Characteristics	Overall (%), *n* = 285
*Age (years)*	
Median (range)	66.8 (41–86)
*Gender, n (%)*	
Female	95 (33.3)
Male	190 (66.6)
*Histology, n (%)*	
Adenocarcinoma	190 (66.7)
Squamous-cell carcinoma	95 (33.3)
*Smoking status, n (%)*	
Never	38 (13.3)
Current	140 (49.1)
Past	107 (37.7)
*ECOG, n (%)*	
0	59 (20.7)
1	170 (59.6)
2+	56 (19.7)
*Type of Treatment, n (%)*	
Chemo-immunotherapy	225 (79)
Only immunotherapy	60 (21)
*Type of Immunotherapy, n (%)*	
Pembrolizumab	203 (71.2)
Ipilimumab plus Nivolumab	82 (28.8)
*PD-L1 values n (%)*	
PD-L1 < 1%	102 (35.8)
PD-L1 ≥ 1%	183 (64.2)
*Subgroups of PD-L1 values n (%)*	
PD-L1 1–49%	62 (33.88)
PD-L1 ≥ 50%	121 (66.12)

Abbreviation: *n*, number; *ECOG*, Eastern Cooperative Oncology Group; Note: Immunohistochemistry was performed on tissue biopsy samples.

**Table 2:** Association Between PD-L1 Expression Levels and Dermatological Toxicities (Rash and Pruritus) in patients.

PD-L1 Values	Rash (*n* = 57) (%)	*p* Value	Pruritus (*n* = 47) (%)	*p* Value
PD-L1 < 1%	20 (19.6)	0.91	9 (8.8)	0.0013
PD-L1 ≥ 1%	37 (20.2)		38 (20.2)	
PD-L1 1–49%	13 (7.1%)	0.083	14 (7.1%)	0.206
PD-L1 ≥ 50%	24 (13.1%)		24 (13.1%)	

Abbreviation: *n*, number; PD-L1; Programmed death-ligand 1.

**Table 3:** Association Between type of treatment and Dermatological Toxicities (Rash and Pruritus).

Type of Treatment	Rash (*n* = 57) (%)	*p* Value	Pruritus (*n* = 47) (%)	*p* Value
only immunotherapy	11 (18.3%)	0.856	11(18.3%)	0.747
chemo-immunotherapy	46 (20.4%)		35 (15.6%)	

Abbreviation: *n*, number.

**Table 4:** Association Between type of Immunotherapy administrated and Dermatological Toxicities (Rash and Pruritus).

Type of Immunotherapy	Rash (*n* = 57) (%)	*p* Value	Pruritus (*n* = 47) (%)	*p* Value
Pembrolizumab	43 (21.1%)	0.181	38 (18.7%)	0.075
Ipilimumab plus nivolumab	11 (13.4%)		8 (9.8%)	

Abbreviation: *n*, number.

**Table 5:** Association Between Gender and Dermatological Toxicities (Rash and Pruritus).

Gender	Rash (*n* = 57) (%)	*p* Value	Pruritus (*n* = 47) (%)	*p* Value
Male	36 (19%)	0.535	19 (22%)	0.118
Female	21 (22.1%)		27 (14.2%)	

Abbreviation: *n*, number.

**Table 6:** Time-Dependent Cox Regression for Overall Survival.

Variable	HR	95% CI	*p*-Value
Rash (time-dependent)	1.12	0.82–1.54	0.47
Pruritus (time-dependent)	0.81	0.58–1.14	0.23
PD-L1 ≥ 1%	0.62	0.48–0.80	<0.001
Age (per year)	1.02	1.01–1.04	0.021
Male sex	1.10	0.84–1.44	0.49
ECOG ≥ 2	1.78	1.29–2.45	<0.001
Squamous histology	1.16	0.89–1.52	0.27
Current smoker	1.34	1.01–1.78	0.043
Chemo-immunotherapy	0.92	0.68–1.25	0.60

Abbreviation: PD-L1; Programmed death-ligand 1; ECOG, Eastern Cooperative Oncology Group; HR, hazard ratio; CI, Confidence Interval.

**Table 7:** Time-Dependent Cox Regression for Progression-Free Survival.

Variable	HR	95% CI	*p*-Value
Rash (time-dependent)	0.97	0.74–1.28	0.84
Pruritus (time-dependent)	0.68	0.50–0.92	0.014
PD-L1 ≥ 1%	0.59	0.46–0.75	<0.001
Age (per year)	1.01	1.00–1.03	0.08
Male sex	1.08	0.84–1.38	0.55
ECOG ≥ 2	1.65	1.23–2.20	0.001
Squamous histology	1.12	0.87–1.44	0.38
Current smoker	1.28	0.98–1.66	0.07
Chemo-immunotherapy	0.95	0.72–1.26	0.73

Abbreviation: PD-L1; Programmed death-ligand 1; ECOG, Eastern Cooperative Oncology Group; HR, hazard ratio; CI, Confidence Interval.

## Data Availability

The data either resides within the article itself or can be obtained from the authors upon making a reasonable request.

## Abbreviations

The following abbreviations are used in this manuscript:

ICIs	immune checkpoint inhibitors
cAEs	Cutaneous adverse events
PD-1	Programmed Cell Death
PD-L1	Programmed Cell Death-1 Ligand
SCLC	Small-cell lung cancer
NSCLC	Non-small-cell lung cancer
SCC	squamous-cell carcinoma
TPS	Tumor Proportion Score
irAEs	immune-related adverse events
DRESS	drug reaction with eosinophilia and systemic symptoms
SJS	Stevens–Johnson syndrome
TEN	toxic epidermal necrolysis
HLA	human leukocyte antigen
OS	overall survival
PFS	progression-free survival
HRs	hazard ratios
CIs	confidence intervals
RECIST	Response Evaluation Criteria in Solid Tumors
ECOG-PS	Eastern Cooperative Oncology Group Performance Status
